# Delivery place preference and its associated factors among women who deliver in the last 12 months in Simada district of Amhara Region, Northwest Ethiopia: a community based cross sectional study

**DOI:** 10.1186/s13104-019-4158-7

**Published:** 2019-03-01

**Authors:** Maru Mekie, Wubet Taklual

**Affiliations:** 1 Department of Midwifery, College of Health Sciences, Debre Tabor University, P.O. Box: 272, Debre Tabor, Ethiopia; 2 Department of Population Health, College of Health Sciences, Debre Tabor University, Debre Tabor, Ethiopia

**Keywords:** Delivery place preference, Home delivery, Determinant factors, Amhara, Ethiopia

## Abstract

**Objective:**

The aim of this study was to assess delivery place preference and its determinant factors in Simada District of Amhara Region, Northwest Ethiopia. Data was collected among 346 women who delivered in the last 12 months.

**Result:**

Of the total 362 study participants, 346 were included in the analysis giving a response rate of 95.6%. More than half, 56.4% of the study participants reported home as their preferred delivery place. The odds of preferring home delivery was higher among women with low household income (AOR = 2.13, 95%, CI (1.06, 4.35)), and those who had < 4 antenatal care visits (AOR = 3.65, 95%, CI (1.58, 8.41)). Whereas, preference of home delivery was lower (AOR = 0.13, 95%, CI (0.05, 0.32)), (AOR = 0.40, 95%, CI (0.17, 0.98)), and (AOR = 0.31, 95%, CI (0.15, 0.67)) among women with facility delivery, within 5 km distance to health facility, and who had transport access respectively. Improving access of health facility to the nearest possible and improving transport access shall be emphasized to improve institutional delivery.

**Electronic supplementary material:**

The online version of this article (10.1186/s13104-019-4158-7) contains supplementary material, which is available to authorized users.

## Introduction

There is pronounced disparity in maternal mortality between developed and developing countries which showed 99% of maternal mortality burden resides in developing countries [1]. The life time risk of maternal mortality is found to be 1 in 41 and 1 in 3300 pregnancies among developed and developing countries respectively. Maternal mortality was reported to be 236 per 100,000 live births in low-income countries and 216 per 100,000 at global level in 2015 [2–4].

Ethiopia is one of Sub-Saharan country where maternal mortality is a great public health concern. Maternal mortality is found to be higher in the country even compared to other low income countries. According to the 2016 Ethiopia demographic and health survey, maternal mortality ratio was 412 per 100, 000 live births. Despite a significant maternal mortality reduction has been achieved from 1400 per 100,000 in 1990 to 412 per 100,000 in 2015, the problem is still un acceptably high in the country [5, 6]. Studies revealed that more than three quarter of maternal mortality is attributed to direct obstetrics causes which could be resolved by skilled delivery care [2, 7].

The government of Ethiopia has been undertaking several measures to counteract maternal morbidity and mortality such as expansion of health facilities, construction of maternity waiting homes, training, and deployment of midwives confined to health sector transformation plan [7, 8].

Despite substantial measures have been undertaken by the Ethiopian government and support organizations [9], institutional delivery is still unacceptably low in the country. Place of delivery is critical factor for women and child survival [10]. Hence, identifying potential determinant factors influencing women’s preference of delivery site is crucial for program implementers and policy makers to achieve the health sector transformation plan [7]. This study aimed to assess determinants of delivery place preference and women’s experience in place of delivery.

## Main text

### Methods

#### Study setting

A community based cross-sectional study design was employed in Simada district of Amhara Region, Northwest Ethiopia from March to May 2018. Women who delivered in the last 12 months and permanent resident of the district were included. Whereas, those who refused to participate and those lived < 6 months in the district were excluded.

#### Sample size and data collection procedures

The sample size was performed by using a single population proportion formula with Z*α*/2 = 1.96, p = 0.31 [11], d (margin of error) = 5%. The final sample size after adding 10% non-response rate was 362. There were 10,112 women who delivered in the last 12 months in the district and the calculated sample was proportionally allocated to kebeles (the lowest administrative scheme in Ethiopia). Then study participants were selected by simple random sampling technique by using the list of women who delivered in the last 12 months as a sample frame. The data was collected by pretested questionnaire (see Additional file 1) adapted from Ethiopian Demographic and Health Survey [5] through face to face interview by trained data collectors. The quality of the data was ensured during data collection, coding, entry, and analysis.

#### Analysis

The data were entered in Epi-data Version 3.1, cleaned, edited, and analyzed by SPSS version 20. Binary logistic regression was performed to identify factors affecting preference of delivery place among study participants. Variables with a p-value of < 0.2 in the binary logistic regression were included in the multivariable model to identify the predictors of preference of delivery place. The statistical significance is confirmed at p < 0.05 with 95% confidence interval.

#### Ethical approval

The ethical issue was approved by Research Review Committee of the College of Health Sciences, Debre Tabor University. An official letter was written to administrative body of Simada district and the administrative bodies of the kebeles. Study participants were informed that the participation is voluntary and confidentiality of the information was maintained.

### Results

#### Socio-demographic characteristics of the study participants

Of the total 362 samples, 346 were included in the analysis giving a response rate of 95.6% in which 16 cases were excluded in the analysis due to incompleteness. The mean age of the study participants was 30.82 years and the standard deviation was ± 6.41 years (see Table 1).

**Table 1:** Socio-demographic characteristics of women who delivered in the last 12 months in Simada district of Amhara region, Ethiopia

Variables	Frequency (N)	Percent (%)
Age (years)		
< 20	19	5.5
20–34	224	64.7
≥ 35	103	29.8
Marital status		
In union	318	91.9
Not in union	28	8.1
Education		
No formal education	183	52.9
Primary education	113	32.7
Secondary and above	50	14.5
Occupation		
House maker	319	92.2
Student	12	3.5
Government employee	15	4.3
Monthly household income (ETB)		
< 1800	161	46.5
≥ 1800	185	53.5
Husband education		
No formal education	168	48.6
Primary education	112	32.4
Secondary and above	66	19.1
Husband occupation		
Government employee	31	9.0
Farmer	287	82.9
Merchant	14	4.0
Daily laborers	14	4.0

#### Obstetrics characteristics and factors influencing preference of delivery place

More than half, 195 (56.4%) of women prefer home delivery. Transport problem and far distance of health facility were reasons mostly cited by respondents with respective frequencies of 55 (28.1%) and 51 (26%). Trust on TBA and unfriendly care accounts for 36 (18.4%) and 25 (12.8%) respectively as a driving factors for preference of home delivery (Table 2.)

**Table 2:** Preference of place of delivery and related factors of women who delivered in the last 12 months in Simada district of Amhara region, Ethiopia

Variables	Frequency (N)	Percent (%)
Woman preference of delivery		
Facility	151	43.6
Home	195	56.4
Last place of delivery		
Facility delivery	150	43.4
Home delivery	196	56.6
Reasons for home delivery		
Short delivery time	29	14.8
Far facility	51	26.0
Transport problem	55	28.1
Trust on TBA	36	18.4
Unfriendly care	25	12.8
Who manage at home		
Mother in low	15	7.7
TTBA	21	10.7
Female neighbor	89	45.4
Health extension workers	4	2.0
Untrained TBA	67	34.2
Decision of delivery place		
Husband/TBA	195	56.4
Woman herself	151	43.6
Distance of health facility (km)		
< 5	182	52.6
≥ 5	164	47.4
Transport access		
Yes	159	46.0
No	187	54.0

#### Pregnancy and child birth related characteristics of the study participants

With regards to number of pregnancies, 81 (23.4%) and 96 (27.7) participants were primiparous and grand multiparous respectively. More than a quarter, 97 (28%) of study participants had no information about advantage of giving birth at a health facility.

Previous institutional delivery and living within 5 km distance to health facility were found to be associated with preference of institutional delivery (Table 3).

**Table 3:** Multivariable analysis of factors determining preference of delivery place among women who delivered in the last 12 months in Simada district, Ethiopia

Variables	Facility delivery N (%)	Home delivery N (%)	COR (95%)	AOR (95%)
Education				
No formal education	62 (41.1)	121 (62.1)	3.79 (1.96, 7.33)*	0.87 (0.18, 4.25)
Primary education	56 (37.1)	57 (29.2)	1.98 (0.99, 3.95)	1.01 (0.26, 3.95)
Secondary and above	33 (21.9)	17 (8.7)	1	1
Monthly income (ETB)				
< 1800	56 (37.1)	105 (53.8)	1.98 (1.28, 3.05)*	2.13 (1.06, 4.35)**
≥ 1800	95 (62.9)	90 (46.2)	1	1
Husband education				
No formal education	56 (37.1)	56 (28.7)	1.75 (0.94, 3.26)	1.25 (0.33, 4.75)
Primary education	53 (35.1)	115 (59.0)	3.80 (2.09, 6.90)*	2.42 (0.57, 10.30)
Secondary and above	42 (27.8)	24 (12.3)	1	1
Previous delivery place				
Facility	108 (71.5)	42 (21.5)	0.11 (0.07, 0.18)*	0.13 (0.05, 0.32)**
Home	43 (28.5)	153 (78.5)	1	1
Decision of delivery place				
Husband/TBA	61 (40.6)	134 (68.7)	3.24 (2.08, 5.05)*	1.11 (0.51, 2.39)
Woman herself	90 (59.6)	61 (31.3)	1	1
Distance of facility (km)				
< 5	99 (65.6)	83 (42.6)	0.39 (0.25, 0.60)*	0.40 (0.17, 0.98)**
≥ 5	52 (34.4)	112 (57.4)	1	1
Transport access				
Yes	90 (59.6)	69 (35.4)	0.37 (0.24, 0.58)*	0.31 (0.15, 0.67)**
No	61 (40.6)	126 (64.6)	1	1
No of past pregnancies				
1 pregnancy	38 (25.2)	43 (22.1)	0.49 (0.26, 0.91)*	0.96 (0.32, 2.86)
2–5 pregnancies	84 (55.6)	85 (43.6)	0.44 (0.26, 0.74)*	0.49 (0.21, 1.55)
> 5 pregnancies	29 (19.2)	67 (34.4)	1	1
Know advantage of facility birth				
Yes	136 (90.1)	113 (57.9)	0.15 (0.08, 0.28)*	0.57 (0.20, 1.63)
No	15 (9.9)	82 (42.1)	1	1
Number of ANC				
< 4 visit	88 (67.7)	105 (89.7)	4.18 (2.07, 8.42)*	3.65 (1.58, 8.41)**
≥ 4 visits	42 (32.3)	12 (10.3)	1	1

In this study, grand multiparity is defined as women who have > 5 deliveries

1 reference

* Significant on binary analysis

** Significant on multivariable analysis

### Discussion

Less than half, 151 (43.6%, 95%, CI 37.9%, 49.2%) women prefer facility delivery as their preferred delivery place. The prevalence of institutional delivery is better than the national average (26%) [5]. However, the finding of our study is found to be much lower than a study conducted in Debre Markos town of Ethiopia [12]. The difference could be related to difference in study population in which the aforementioned study was conducted in urban area unlike our study.

With regards to reasons of preference of home delivery; transport problem and far distance of health facility were reasons mostly cited by respondents with respective frequencies of 55 (28.1%) and 51 (26%). Trust on TBA and unfriendly care accounts for 36 (18.4%) and 25 (12.8%) respectively as a driving factors for preference of home delivery. The finding is consistent with previous studies [13, 14]. Government shall work on compassionate respectful maternity care in addition to improving the competency of health care workers to increase women’s trust [7].

With regards to previous place of delivery, our study revealed that the odds preferring home delivery was found to be lower among women who delivered at health facility in previous births compared with counterparts (AOR = 0.13, 95%, CI (0.05, 0.32)). The finding of our study is supported by previous studies [12, 15]. Similarly, the odds of preferring home delivery was found to be 2.13 times higher among women with household income of < 1800 ETB (AOR = 2.13, 95%, CI (1.06, 4.35)). The finding is supported by previous studies [14, 16].

With regards to distance to health facility, the odds of preferring home delivery was found to be lower among women living within 5 km distance (AOR = 0.40, 95, CI (0.17, 0.98)) compared with counterparts. Women who live > 5 km distance to health facility might face difficulty of transport access, cost and other related logistics which are likely to be associated with preference of home delivery. The finding of our study is consistent with previous studies [14, 17, 18]. Likewise, the odds of preferring home delivery was found to be lower among women who had transport access (AOR = 0.31, 95%, CI (0.15, 0.67)) compared with counterparts. The finding of our study is consistent with previous studies [17, 19, 20].

Consistent with previous studies [13, 17], the odds of preferring home delivery was found to be 3.7 times higher among women who had < 4 ANC visits (AOR = 3.65, 95%, (1.58, 8.41)) compared with women who had ≥ 4 ANC visits. This might be women had frequent ANC visit had a chance to be counseled frequently about delivery place, birth preparedness, and complication readiness plan.

### Conclusion and recommendation

Institutional delivery in the study district was better than the national average. Women who had low household income and less than 4 ANC visit were more likely to prefer home delivery. Whereas, women who deliver at health facility in previous pregnancy, had transport access, and within 5 km radius to health facility were more likely to prefer institutional delivery. Improving transport access and health facility to the nearest possible shall be emphasized. Lessons that could be taken from this study are; apart from improving health and transport infrastructure, the influence of poor quality of health care cannot be undermined.

## Limitations

The study was conducted in one district which might affect generalizability and representativeness of the finding. Cross-sectional study design was used which might affect cause effect relationship. However, effort was made to increase generalizability by collecting data at community level and those who delivered in the last 12 months to minimize recall bias which can be taken as a strength.

## Additional file

**Additional file 1.** English version Questionnaires used to assess delivery place preference.

## Abbreviations

ANC: antenatal care
ETB: Ethiopian Birr
TBA: traditional birth attendant
TTBA: trained traditional birth attendant
