# Supplementary material for: Delivery place preference and its associated factors among women who deliver in the last 12 months in Simada district of Amhara Region, Northwest Ethiopia: a community based cross sectional study
Source: BMC Res Notes. 2019 Mar 1;12:114. doi: 10.1186/s13104-019-4158-7 (PMC6397443; doi:10.1186/s13104-019-4158-7)
Supplement: Supplementary file 1 — Additional file 1. English version Questionnaires used to assess delivery place preference. [file 13104_2019_4158_MOESM1_ESM.docx]

Name of the interviewer: ______________Sign. _____ Date of interview________

Name of the supervisor: _____________ Sign. _______ Date________

**Identification in particular**

1) Households Identification

001. Questionnaire Code______

002. Woreda___________

003. Residence 1. Rural___________ 2. Urban ____________

004. How long have you been living in this place? _____Years

**Part I: Maternal socio demographic characteristics**

| Sr.No | **Questions** | Answer to questions |
| --- | --- | --- |
| 101 | What is your age | In years................. |
| 102 | What is your marital status | A. Married B. Divorced C. Widowed  D. Single E. Separated F. Cohabited |
| 103 | What is your religion | A. Orthodox B. Muslim  C. Protestant D. Others(specify) ____________ |
| 104 | What is your ethnicity | A. Amhara, B. Oromo,  C. Tgray D. Others, specify---------- |
| 105 | What is your occupation | A. House wife B. Governmental Employee  C. Merchant D. Daily Labourer  E. Student. F. Other specify....... |
| 106 | What is your educational Status | A. no formal education  B. Primary education(1-8)  C. Secondary education(9-12)  D. College or University |
| 107 | What is your monthly household income | ------------------------Ethiopian Birr |
| 108 | What is your husband educational Status | A. no formal education  B. Primary education(1-8)  C. Secondary and above  D. College or University |
| 109 | What is your husband’s occupation | A. Farmer. B. Governmental employee  C. Merchant D. Daily labourer  E. Other, specify---------------- |

**.Part II: Women’s choice on place of delivery and the factors for their choice.**

| No | Question | Option | Skip to  question | Code |
| --- | --- | --- | --- | --- |
| 201 | Where is your choice regarding to your place of delivery? | 1. Health institution  2. Home | If (health  institution  n) skip to  ques. 203 |  |
| 202 | If no (1)is home:  What is your main reason to prefer home delivery?  (MORE THAN ONE ANSWER POSSIBLE) | 1. Distance of health institution is far from my home  2. No means of transportation  3. I have no money to pay for transport and health service  4. I dislike the behavior of health workers  5. Trust on TBA  6. The service is not available  7. Not necessary to go to Health Inst. for labor & delivery  8. Because my culture/religion restrict me  9. Facility not open regularly  10. Poor quality service of HFs  11. No female provider at HFs  12. Husband will not allow me  13. Labor was smooth and short  66. Other (specify)__________ |  |  |
| 203 | If no (1) is HI: what is your main reason to prefer health institution delivery?(MORE THAN ONE)  ANSWER POSSIBLE | 1. Better service  2. Safe and clean delivery  3. Close to my home  4. I was informed to deliver in health institution  5. Antenatal attendance at the  Health Inst.  6. Fear of complications  7. HCWs offer friendly services  66. Other (specify)__________ |  |  |
| 204 | Where did your last delivery take  place | 1. Health institution  2. Home | If 1 go to  ques.20 8 |  |
| 205 | Did you plan to deliver at home? | 1. Yes 2. No |  |  |
| 206 | If you delivered at home who assisted you? | 1. Mother  2. Mother –in-low  3. TTBA  4. Women from my Neighbor  5. Health extension workers  6. TBA  66.Other(specify)__________ |  |  |
| 207 | Is there any traditional medication given to the mother during child birth at home? | 1. Yes  2. No | If 2  Go to  Quest. 208 |  |
| 208 | What is the reason for providing the medication? | 1. To hasten child birth  2. To relief pain  3. To prevent complication of child birth  66. Other (specify)__________ |  |  |
| 209 | If at health facility who assisted you? | 1. Health extension workers.  2. Nurse  3. Midwife  4. Health officer  5. Doctor  88. Don't remember |  |  |
| 210 | Who decides on place of your delivery? | 1. Just me  2. My husband  3. Both My husband and me  4. TBA  5. My Mother and mother in law  66. Other (specify)__________ |  |  |
| 211 | Where is the choice of your husband regarding your place of delivery? | 1. Health institution  2. Home |  |  |
| 212 | Where will your next delivery, when you are pregnant/ if you are pregnant now? | 1. Health institution  2. Home |  |  |
| 213 | Is the road accessible for transportation? | 1. Yes 2. No |  |  |
| 214 | What is the estimated distance from home to the nearby delivery institution? | 1. below 2km  2. 2-5km  3. 5-10 km  4. >10 km |  |  |
| 215 | What means of transport do you use to reach the health facility? | 1. On foot  2. Vehicle  3. Other (specify)__________ |  |  |
| 216 | How long would it take you to reach the health facility from your home? | 1. Less than 1 hour  2. 1 – 2 hours  3. 3 – 4 hours  4. Over 5 hours |  |  |

**III. Women’s past obstetrical history**

| NO | Question | Option | Skip to question | code |
| --- | --- | --- | --- | --- |
| **301** | Age at first pregnancy? | ----------------------- |  |  |
| **302** | Gravidity/total number of pregnancy | 1. 1  2. 2-5  3. >5 |  |  |
| **303** | Parity/total number of births | 1. 1  2. 2-5  3. >5 |  |  |
| **304** | Do you have any information about the benefit of delivery in health institution? | 1. Yes  2. No | If 2  skip to q  307 |  |
| **305** | If yes what is the primary source of information | 1. Health workers  2. Friends, neighbors who get  similar service  3. Media like TV/Radio  66. Other (specify)__________ |  |  |
| **306** | Did you attend antenatal care during your last child pregnancy? | 1. Yes  2. No | If No  skip to q  309 |  |
| **307** | If yes how many visits you have for antenatal? | 1. 1 2. 2-3 3. 4 and more |  |  |
| **308** | Had you developed any obstetric difficulties in previous delivery?  (prolonged labor, hemorrhage) | 1. Yes  2. No |  |  |
| **309** | If yes, What specific measures were taken? | 1. Nothing  2. Visited health institution  3. Massage, herbs, taking different soft drinks  66.Other (specify)__________ |  |  |
